# Rare neurodevelopmental conditions and parents’ mental health – how and when does genetic diagnosis matter?

**DOI:** 10.1186/s13023-024-03076-2

**Published:** 2024-02-15

**Authors:** Zhaotian Chi, Rory T. Devine, Jeanne Wolstencroft, David Skuse, Claire Hughes, Kate Baker, IMAGINE-ID consortium

**Affiliations:** 1 MRC Cognition and Brain Sciences Unit, University of Cambridge, 15 Chaucer Road, CB2 7EF Cambridge, UK; 2 School of Psychology, University of Birmingham, Birmingham, UK; 3 UCL Great Ormond Street Institute of Child Health, London, UK; 4 Centre for Family Research, Department of Psychology, University of Cambridge, Cambridge, UK; 5 Department of Medical Genetics, University of Cambridge, Cambridge, UK

**Keywords:** Intellectual disability, Parents, Carers, Mental health, Genetic

## Abstract

**Background:**

Parents of individuals with rare neurodevelopmental conditions and intellectual disabilities (ID) are vulnerable to mental health difficulties, which vary between parents and within parents over time. The underlying cause of a child’s condition can influence parents’ mental health, via uncertain pathways and within unknown time-windows.

**Results:**

We analysed baseline data from the IMAGINE-ID cohort, comprising 2655 parents of children and young people with ID of known genetic origin. First, we conducted a factor analysis of the SDQ Impact scale to isolate specific pathways from genetic aetiology to parents’ mental health. This suggested a two-factor structure for the SDQ Impact scale, with a “home & distress” dimension and a “participation” dimension. Second, we tested via structural equation modelling (SEM) whether genetic diagnosis affects Impact and mental health directly, or indirectly via children’s characteristics. This analysis identified an indirect pathway linking genetic aetiology to parents’ mental health, serially through child characteristics (physical disabilities, emotional and behavioural difficulties) and Impact: home & distress. Third, we conducted moderation analysis to explore the influence of time elapsed since genetic diagnosis. This showed that the serial mediation model was moderated by time since diagnosis, with strongest mediating effects among recently diagnosed cases.

**Conclusions:**

There are multiple steps on the pathway from ID-associated genetic diagnoses to parents’ mental health. Pathway links are strongest within 5 years of receiving a genetic diagnosis, highlighting opportunities for better post-diagnostic support. Recognition and enhanced support for children’s physical and behavioural needs might reduce impact on family life, ameliorating parents’ vulnerabilities to mental health difficulties.

**Supplementary Information:**

The online version contains supplementary material available at 10.1186/s13023-024-03076-2.

## Background

Intellectual disability (ID) is defined as childhood-onset impairment in cognitive and adaptive function, and affects 1–3% of the worldwide population [1–3]. Parents of children and young people (hereafter children) with ID are at risk of reduced well-being and increased mental health symptoms [4–6]. Compared with parents of typically developing children, parents of children with ID have higher levels of depression [7], anxiety [8] and somatic symptoms [9]. Parenting a child with ID can entail risk to mental health for several reasons. Firstly, a child’s neurodevelopmental differences and associated medical problems may place substantial strain on parents’ emotional well-being [10, 11]. Children with ID are more likely to have physical health problems and prolonged caring needs in relation to mobility, sleep and personal care, and are also more likely to experience internalising and externalising behavioural problems [12, 13]. These multiple stressors can adversely affect parental well-being. Secondly, parents of children with ID are more likely to face socio-economic disadvantage, which can contribute to increased risk of mental health problems [14, 15]. Thirdly, having a child with ID may be a non-specific stress factor interacting with parents’ intrinsic characteristics such as coping style, personality and physical health [16]. Fourthly, having a child with ID can be intrinsically emotionally challenging, calling into question hopes and expectations of the future for oneself and one’s family. Irrespective of these complementary contributors to parents’ mental health, it is important to recognise and respond to parents’ needs, because of the effects on parents themselves, on their children (with and without ID), and their wider families and social circles.

The challenges faced by parents of children with ID have long been recognised. However, one aspect of families’ experience that is changing is the increased availability (in economically advantaged circumstances) of diagnostic genetic testing. Until recently, most ID was of unknown cause, resulting in lifelong uncertainties for parents such as whether ID could have been prevented, whether ID will affect future generations within a family, and whether a child’s condition will improve or worsen with time. Next Generation Sequencing (NGS) technologies can now identify a causal diagnosis in over 40% of individuals with ID [17], and 60% of individuals with severe ID [18]. In tandem with this increase in diagnostic yield, more than 2500 genes have been associated with ID [19]. Genetic diagnosis can reduce (though not abolish) parents’ uncertainties, bringing emotional relief and a better understanding of medical needs [20–24]. However genetic diagnosis is often accompanied by new questions about an individual’s prognosis, treatment, and supportive management to maximise positive outcomes. Progress in genetic diagnosis amplifies research questions relating to parents’ well-being: Do differences in the specific genetic aetiology of a child’s ID contribute to variation in parent’s mental health risks? How does the genetic diagnostic process affect parents’ psychological well-being? Does the influence of genetic diagnosis on parental well-being change over time?

Existing evidence suggests that parents’ experiences differ according to the ID-related genetic diagnosis of their child. For example, comparison between parents of children with Down syndrome, Rett syndrome and CDKL5-associated disorder found that mothers of children with CDKL5-associated disorder, associated with high medical needs and poor neurodevelopmental prognosis, had poorest well-being [6]. Similarly, in a study of 13 genetic syndrome groups, specific aetiology contributed to variation in parental depression symptoms [25]. In the largest study to date exploring the association between genetic diagnoses and caregiver mental health, Baker et al. (2020) analysed data from the first 888 families participating in the IMAGINE-ID study [26]. This cohort encompasses the extreme heterogeneity of rare and ultra-rare genomic variants that can now be diagnosed via chromosome microarray analysis (copy number variants, CNVs; chromosome rearrangements) and NGS (single nucleotide variants, SNVs). Results were consistent with other studies regarding important non-genetic factors: the strongest influences on parental well-being were recent life events affecting a family, and parental appraisal of the impact of children’s difficulties [14, 27]. Impact was itself predicted by child age, physical disability, autistic characteristics, and behavioural difficulties. A novel observation was that the type of genetic diagnosis, broadly categorised into CNVs, SNVs and chromosomal disorders, also influenced impact appraisal - CNV diagnoses were associated with elevated impact, not explained by CNV inheritance, neighbourhood deprivation or family structure.

These previous results leave at least three questions to be addressed. Firstly, except for life events, all predictive factors (including type of genetic diagnosis) affect well-being indirectly via parental appraisal of impact. But what is “impact”? By definition, it refers to caregivers’ or teachers’ subjective evaluation of difficulties, reflecting chronicity, distress, social impairment and burden [28]. Distinction should be drawn between functional impact on a child’s daily life and activities (e.g., effect on school participation), and family impact (e.g., effect on sibling relationships), which were not examined separately in previous analysis [26]. The second gap concerns mediating relationships between genetic diagnosis, children’s characteristics, and parental well-being; the pathways and processes through which genetic diagnosis exerts an effect are unknown. The third gap concerns the timing of genetic diagnosis, which could potentially confound the observed effect of genetic diagnosis or could moderate relationships between genetic diagnosis and parental psychology. The current paper addresses these three questions.

## Methods

### Cohort recruitment, data collection and sample inclusion criteria

Recruitment to the IMAGINE ID study and data collection methods are described in [29]. In brief, participants were recruited between 2014 and 2020 from UK-wide Regional Genetics Centres, other research cohorts, social media, and support groups. Inclusion criteria were (A) the index child had been diagnosed with ID or developmental delay by a specialist physician, (B) the child had received a molecular diagnostic result (one or more identified CNV or SNV, or other genetic diagnosis, likely to cause or contribute to child’s ID) from an accredited diagnostic laboratory, (C) the genetic diagnosis had been communicated to the family via clinical pathways and (D) the child was aged above 3 years when recruited to the study (in line with lower age limit of standardised phenotyping tools). A carer of the child (91.3% mothers, 6.6% fathers, and 2.0% other relatives) completed the data collection process online, by telephone or in person. Data for the present analysis were accessed after project approval from the study committee. Participants aged over 18 years were excluded. Where more than one sibling within the same household had been recruited to the cohort, information about the oldest child within the family was analysed. For a flow chart of the data cleaning process, see Supplementary Material A. The initial sample for factor analysis consists of 2655 participants. For SEM analyses, participants with incomplete or incompatible time since diagnosis information were removed i.e. those without a genetic report or time since diagnosis < 0 (sample size 2423). For moderation analysis focusing on the comparison between SNV and CNV groups, other genetic groups were excluded (sample size 1943).

### Measures

Parents’ well-being and distress was measured by the Everyday Feeling Questionnaire (EFQ), a ten-item measure rated on a five-point Likert scale [30]. The EFQ scale was estimated as a latent variable, combining a common factor and a method factor, to capture the residual common variance caused by positively and negatively scored items [31]. Appraisal of impact was measured by the Strengths and Difficulties Questionnaire Impact Supplement (SDQ-Impact) [32], a five-item subscale measuring the presence of distress or impairment in family life, friendships, learning, and leisure activities. Children’s developmental and behavioural characteristics were assessed by the Developmental and Well-being Assessment (DAWBA) [33]. Children’s developmental quotient (DQ) was estimated by dividing the parental estimate of current mental age by chronological age. A scale for children’s physical disability was constructed from 8 DAWBA items enquiring about toileting, speech, vision, hearing, movement, and seizures (Mean = 2.50, SD = 1.92, ordinal α = 0.67). Children’s social, emotional, and behavioural characteristics were summarised from the DAWBA algorithm-generated binary scores of 70% likelihoods of ICD-10 diagnoses for (A) autism spectrum disorder (ASD), (B) conduct disorder or oppositional defiant disorder (CD/ODD), and (C) any other emotional or behavioural diagnosis (other EBD; separation anxiety, specific phobia, social phobia, OCD, generalized anxiety, depression, ADHD, tic disorder). The likelihood for CD or ODD was combined as they have low diagnosis rate in the sample, they are reported to share some dispositional and environmental risk factors, and ODD can be a precursor to CD [34]. Participants’ socioeconomic status (SES) was estimated by the Index of Multiple Deprivation (IMD) [35], a measure of neighbourhood deprivation. Primary respondents also completed a Negative Life Events checklist, asking about specific events in the past 12 months. Household structure variables (number of children and number of adults resident in the home), were recorded using UK Office for National Statistics definitions. As these two variables are not linearly correlated with dependent variables [36, 37], two binary variables were created to reflect whether the household includes (A) more than one adult or (B) more than one child.

### Genetic diagnosis categorisation and timing

The type and number of children’s genomic variants were categorised based on their diagnostic genetic reports into seven types: (A) CNV, (B) Multiple CNV, (C) SNV, (D) Other chromosomal abnormality (i.e., aneuploidy, translocation or other rearrangement not related to sex chromosomes), (E) Sex chromosome aneuploidy, (F) CNV and SNV, and (G) Multiple SNV. For the analysis of the moderating effect of the time elapsed since genetic diagnosis, the time between age at genetic diagnosis (from laboratory report) and participation in the IMAGINE-ID study was calculated.

### Statistical analysis

Demographic and descriptive data were compared between genetic diagnosis groups using ANOVA and Chi-Square tests, corrected for multiple comparisons (Bonferroni-corrected threshold for significance 0.003).

Factor analysis was conducted to examine the dimensional structure of the SDQ Impact scale within this population, using exploratory factor analysis (EFA) parallel analysis and confirmatory factor analysis (CFA) in R with packages *paran* and *lavaan*. Parallel analysis was first applied to compare the eigenvalues calculated within the target data versus those within random datasets simulated based on the number of observations and items [38, 39]. In this way, factors with higher eigenvalues in the real sample than the generated samples are retained. Alternative factor structures were then compared by confirmatory factor analysis (CFA), using weighted least square (WLS) solution.

The confirmed two-factor latent structure for Impact was then integrated into SEM analyses within the *Lavaan* package in R [40] to examine pathways from genetic aetiology, child characteristics and family background to parents’ EFQ. Two models were tested, respectively examining the effect of genetic diagnosis, and its timing, directly on parents’ mental health (Model A) and indirectly through children’s physical and behavioural characteristics (Model B). Impact dimensions and EFQ were treated as latent variables. Goodness of fit indices include comparative fit index (CFI), Tucker-Lewis Index (TLI), and root-mean-square error of approximation (RMSEA). Threshold of a good fit is defined as CFI and TLI close to 0.95, and RMSEA close to 0.06 [41].

Lastly, a moderation analysis was carried out to examine whether the pathways identified in Model B differ according to time since genetic diagnosis. In this step, only the SNV and CNV groups were included, as the sample was by nature unequally distributed and other groups had relatively low sizes for moderation analysis. The independent variable (SNV or CNV diagnosis) and moderator (Time since diagnosis) were mean centred for the purpose of the analysis. All the predictors in Model A and B were also included in the moderation analyses, to reduce any bias caused by confounding variables. In the last step of the analysis, the moderating effect of time since diagnosis was tested within the moderation models. Bootstrapping method was used to estimate the model parameters [42].

## Results

### Descriptive data

Table 1 describes the full sample included in the factor analysis. For details of each genetic diagnosis group and comparison between groups, see Supplementary Material B. Significant differences between groups were found for child age, gender, DQ, physical disabilities, CD/ODD likelihood, other EBD likelihood, IMD and timing of genetic diagnosis. Groups did not differ in SDQ Impact, parental EFQ scores, ASD likelihood, negative life events, or household membership.

**Table 1:** Descriptive analyses

Continuous		Mean	SD
	Age	9.09	3.82
	DQ	0.53	0.26
	Physical disabilities	2.5	1.92
	Impact	5.43	3.01
	Life events	0.81	1.03
	IMD	5.61	2.92
	EFQ	16.79	7.34
	Time since diagnosis	2.73	3.02
	Age at diagnosis	6.3	4.01
**Categorical**		**N**	**Percentage**
Gender	male	1456	55%
	female	1199	45%
Genetic variant type	CNV	1525	57%
	SNV	583	22%
	Multiple CNV	271	10%
	Other chromosomal	108	4%
	Sex chromosome aneuploidy	93	4%
	CNV and SNV	18	1%
	Multiple SNV	57	2%
ASD likelihood binary	Yes	454	19%
	No	1966	81%
CD/ODD likelihood binary	Yes	458	17%
	No	2197	83%
Other Emotional and Behavioural likelihood binary	Yes	609	23%
	No	2046	77%
More than One adult in household	Yes	1217	88%
	No	165	12%
More than ONE child in household	Yes	950	68%
	No	437	32%

### SDQ impact factor analysis

Polychoric correlations were examined between the five tested items (see Supplementary Material C). Significant correlations were found among all items and especially between classroom learning and friendship, leisure activities and home life, leisure activities and friendship. Supplementary Material D illustrates the parallel analysis comparing factor structure within the dataset to simulated results. Two factors were retained with eigenvalues higher than 0. Exploratory factor analyses (EFA) and confirmatory factor analysis (CFA) for one-factor and two-factor solutions were then compared (Table 2). Factor loadings range from 0.52 to 0.97 in the two-factor model, and from 0.55 to 0.82 in the one-factor model. In the two-factor model, factor 1 is mainly defined by p3 (friendship), p4 (classroom) and p5 (leisure activities), interpreted as impact on participation. P1(upset and distress) and p2(home life) loaded highly on Factor 2, indicating significant convergence on home life and distress. Most items had higher loadings within the two-factor model, except for the distress question which had slightly higher loading (Δ = 0.03) in the one-factor model. The two factors together explained 59% of the variances, whereas the one-factor model explained 51%. Model fit for the two-factor solution is better than the one-factor structure. The two factors have a strong correlation (*r* = 0.64). The second factor has a higher communality in the two-factor solution (0.93) than in the one-factor solution (0.54), which suggests that extra information can be drawn from a factor 2. In both models, impact factor(s) have significant factor variance. CFA results of the two-factor model showed similar goodness of fit indices compared with the previous one-factor model within typically developing children (28), indicating that in this higher risk sample, the scale might have extracted more information than in a low risk sample.

**Table 2:** SDQ Impact factor analysis

	Two-factor solution		One-factor solution
	Factor1	Factor2	Factor 1
EFA Loadings			
p1 Distress		0.57	0.55
p2 Home		0.93	0.74
p3 Friends	0.74	0.10	0.78
p4 Classroom	0.85	-0.11	0.67
p5 Leisure	0.57	0.28	0.81
Proportion Variance	0.32	0.26	0.51
Cumulative Variance	0.33	0.58	NA
CFA model fit	CFI	0.98	0.92
	TLI	0.94	0.85
	RMSEA	0.089	0.14
	SRMR	0.02	0.05

Legend EFA = exploratory factor analysis, CFA = confirmatory factor analysis. Model indices CFI = comparative fit index, TLI = Tucker-Lewis index, RMSEA = root mean square error of approximation, SRMR = standardized root mean square residual

### SEM pathway modelling

**Fig. 1:**
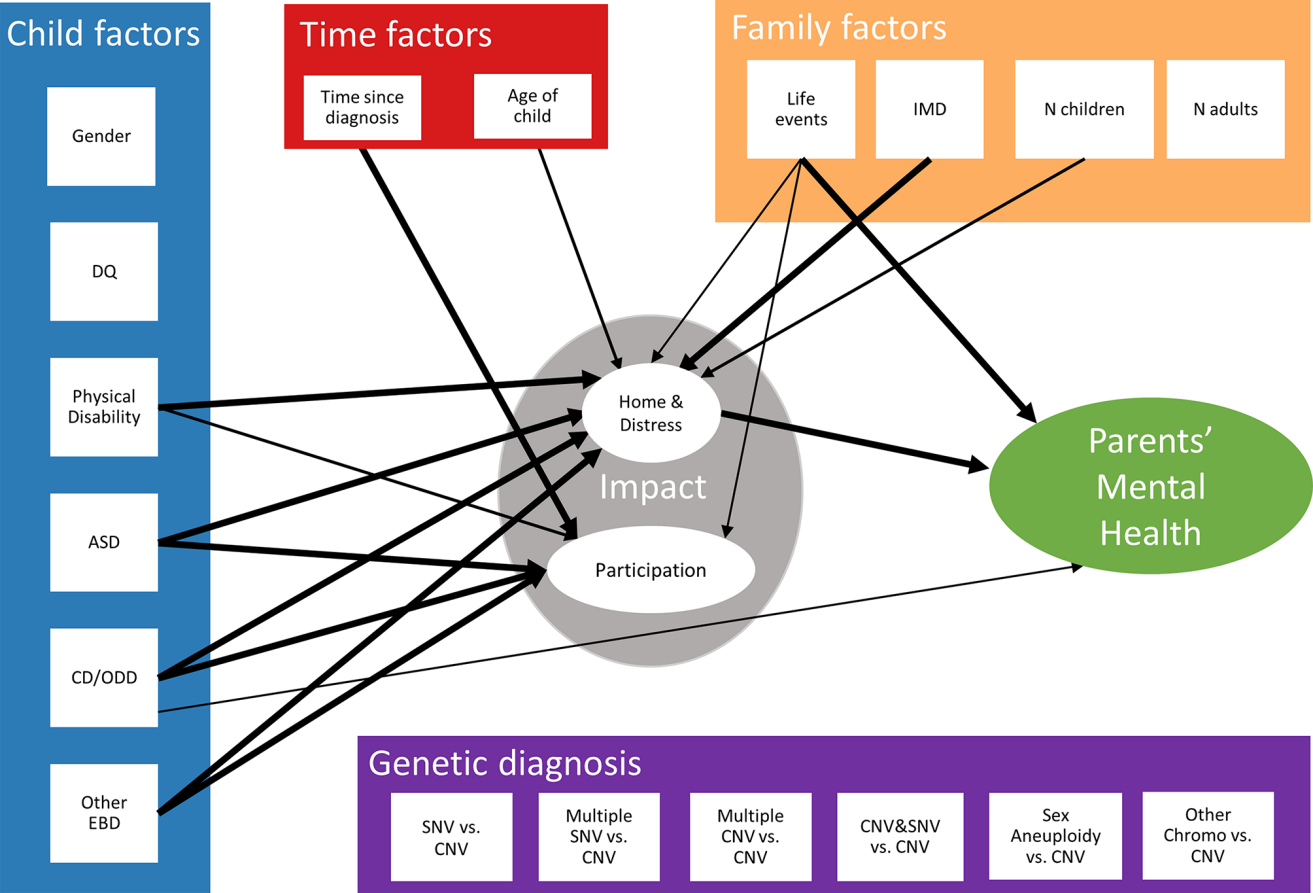
Pathway model A. DQ = developmental quotient, ASD = autism spectrum disorder likelihood, CD/ODD = conduct disorder or oppositional defiant disorder likelihood, Other EBD = other emotional or behavioural disorder likelihood, IMD = Index of Multiple Deprivation decile score, N Children = whether more than one child in household, N Adults = whether more than one adult in household. Thin arrow *p* < 0.05, Medium arrow *p* < 0.01, Thick arrow *p* < 0.001

We then applied the two factor Impact structure to compare two competing pathway models. Figure 1 (Supplementary Material E) summarises results of model A, where genetic variant types were included as direct predictors of Impact dimensions and parents’ mental health. The model fits the data well (CFI = 0.95, RMSEA = 0.03). Child physical (β_1_ = 0.11, β_2_ = 0.12, *p* < 0.001) and behavioural problems (β = 0.11 ~ 0.30, *p* < 0.001) were associated with both Impact dimensions. IMD and family structure (whether child has a sibling) directly related to Impact: home & distress, while negative life events was associated with Impact: home & distress, Impact: participation, and EFQ. Older child age significantly predicted Impact: home & distress (β = 0.08, *p* = 0.004) but not Impact: participation. Longer time since diagnosis, on the other hand, was associated with lower Impact: participation, but not Impact: home & distress. Impact: home & distress was associated with parental mental health (β = 0.30, *p* < 0.001). Genetic diagnosis type did not significantly predict either Impact dimension or parents’ mental health within this model.

**Fig. 2:**
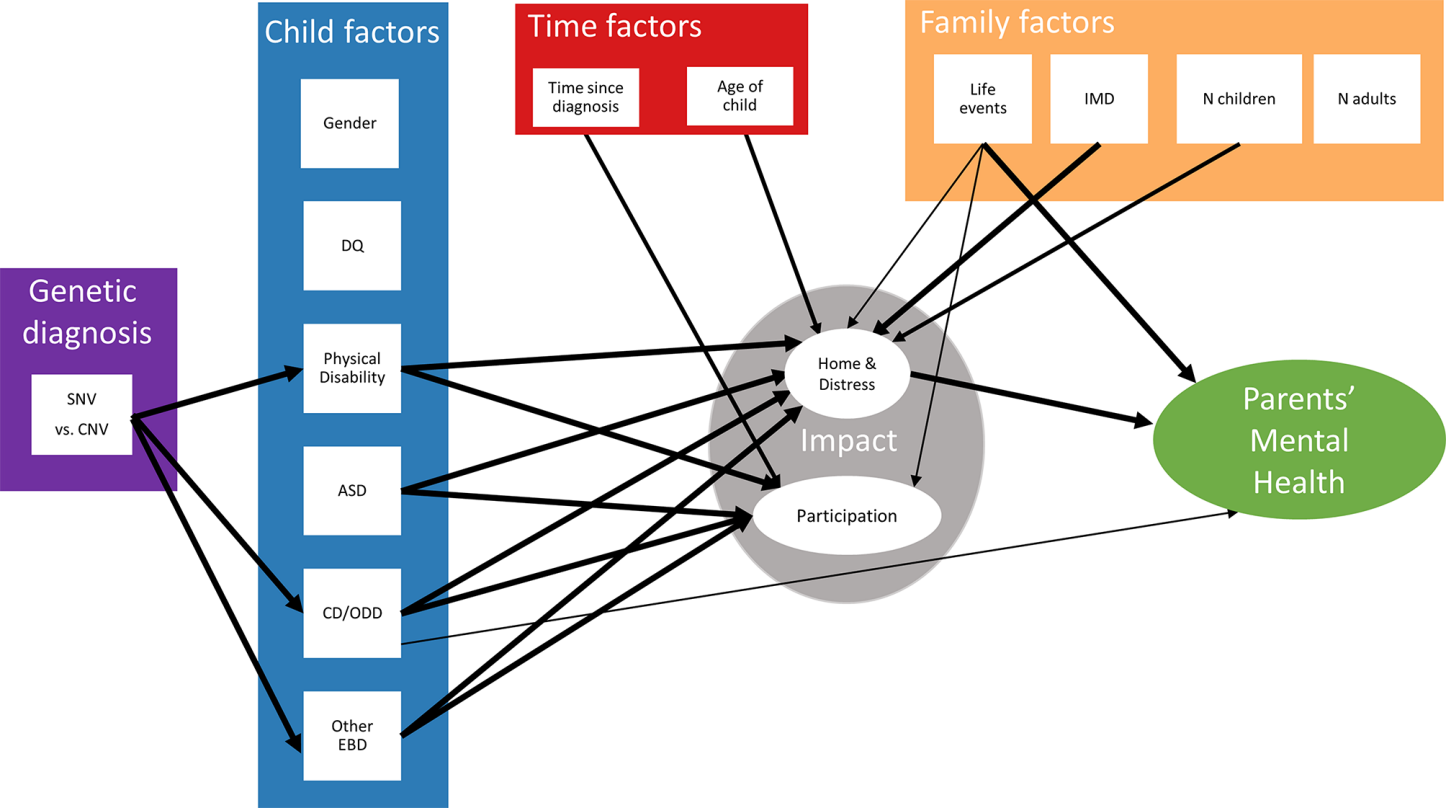
Pathway model B. DQ = developmental quotient, ASD = autism spectrum disorder likelihood, CD/ODD = conduct disorder or oppositional defiant disorder likelihood, Other EBD = other emotional and behavioural disorder likelihood, IMD = Index of Multiple Deprivation decile score, N Children = whether more than one child in household, N Adults = whether more than one adult in household. Effect of all genetic diagnosis types were tested (vs. CNV) but due to limitation of sample sizes in each group, only the effect size of SNV vs. CNV is given. Thin arrow *p* < 0.05, Medium arrow *p* < 0.01, Thick arrow *p* < 0.001

Model B tested the indirect pathways between genetic diagnosis and parental mental health (Fig. 2, Supplementary Material F). The model had acceptable fit (CFI = 0.93, RESEA = 0.04). Again, life events predicted home & distress, participation, and parental mental health, and IMD was directly associated with home & distress. Consistent with model A, age of the child positively related to Impact: home & distress, while time since diagnosis negatively predicted Impact: participation. Focusing on the comparison between SNV and CNV groups (making up the largest proportion of the sample), serial mediating effects were examined for genetic diagnoses, child characteristics, Impact and parental mental health. This indicated a significant total mediation via three child characteristic pathways: significant indirect effects of CD/ODD (Indirect effect = − 0.008, *p* = 0.002), other EBD (Indirect effect = − 0.006, *p* = 0.004) and physical disabilities (Indirect effect = 0.004, *p* = 0.011) on parental mental health via Impact:home & distress. Direct effect of SNV versus CNV on parental mental health is not significant (Direct effect = 0.02, *p* = 0.40), suggesting that the genetic diagnosis effect was explained by the mediating pathways.

### Moderating effect of time since genetic diagnosis

**Fig. 3:**
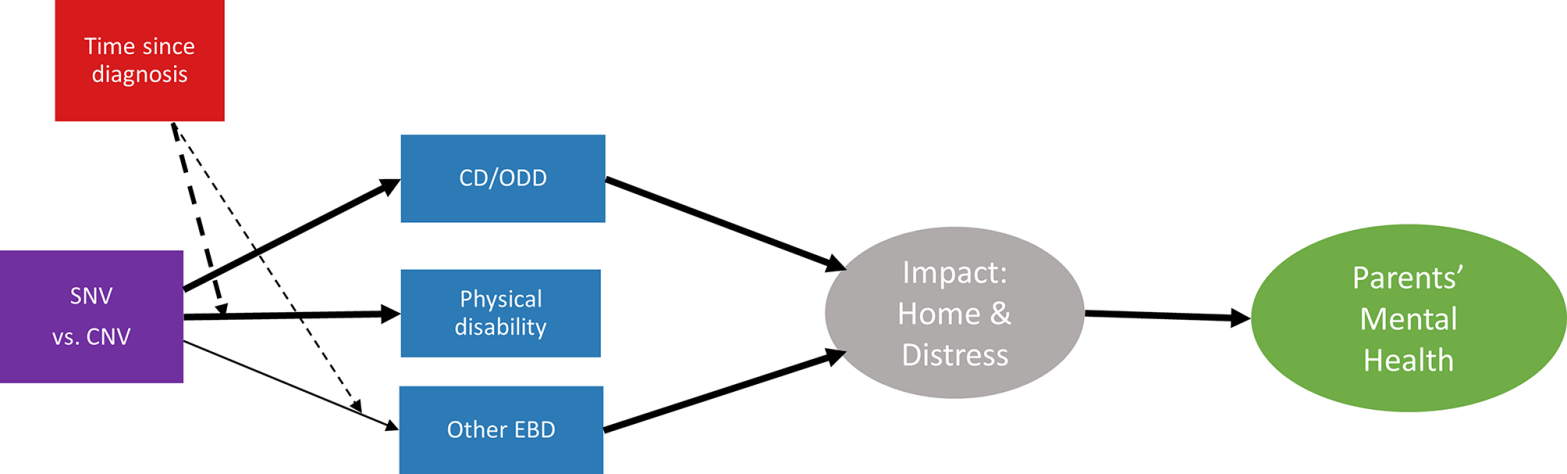
Moderation model. CD/ODD = conduct disorder or oppositional defiant disorder likelihood, Other EBD = other emotional or behavioural disorder likelihood. Hard arrows = significant pathway effects within mediation model. Dashed arrows = significant moderating effects. Thin arrow *p* < 0.05, Medium arrow *p* < 0.01, Thick arrow *p* < 0.001

We further evaluated whether the genetic variant type and time elapsed since diagnosis may interactively affect parental mental health within the above mediation model (Fig. 3, Supplementary Material G). Time since diagnosis (DT) played a moderating role in the direct association between SNV/CNV and physical disabilities (DT * SNV: β = − 0.09, *p* = 0.033), and in the mediating pathway through likelihood of other EBD (DT * SNV: β = 0.11, *p* = 0.005). In both cases, the effect of genetic diagnosis was maximal for recently diagnosed children. Time since diagnosis did not play a moderating role in the association between SNV/CNV and CD/ODD.

## Discussion

We explored variation in parents’ mental health within a large and well-characterised childhood ID cohort. Building on previous observations for the first wave of families recruited to the cohort, we investigated how and when genetic diagnosis might contribute to parents’ emotional state. We confirmed that perceived impact of their child’s condition is a critical convergence point for numerous predictors of parents’ mental health, and we fractionated impact into two dimensions (home&distress and participation) to improve pathway modelling. We found an effect of genetic diagnosis type on parents’ well-being, indirectly via child behavioural and physical characteristics and perceived impact on home&distress. We also explored the possibility of critical time windows during which relevant processes may operate. We found moderating effects of the time since receipt of genetic diagnosis on the serial mediation model via emotional and behavioural difficulties. Whilst these results are cross-sectional, and time-related observations should not be interpreted as developmental, observations suggest a likely dynamic pathway, entailing potential windows of opportunity to enhance support and improve family outcomes.

Stringaris and Goodman (2013) reported a one-factor structure for Impact within the TD population, carrying lower behavioural symptom scores and lower Impact ratings than the ID population. With higher emotional and behavioural difficulties, as well as co-occurring developmental cognitive impairments and physical disability risks, higher parental ratings of impact are expected. We predicted and found a multi-dimensional structure to the Impact scale within the ID population. This aligns with SDQ problem behaviour subscales (not used in the current study), for which two subscales are extracted in low-risk populations but four subscales in high-risk samples [43]. SEM analysis showed that differentiation into the two Impact dimensions provided additional information on pathways toward parents’ well-being. In Model B, which incorporated the greatest number of significant predictor variables, only Impact:home&distress and not Impact:participation acted as a mediator and predicted parents EFQ score. It should be noted that, to date, there is no clear evidence as to whether the SDQ Impact scale measures family impact or functional impact, and correlation with additional measures would improve interpretation of the factor analysis. Given the centrality of impact as a mediator within pathways to parents’ mental health, and impact’s potential as an intervention target [44], further analyses with longer and more specific measurements of functional impairment and family impact will be helpful to further address this gap.

Previously, we found that genetic diagnosis type influenced Impact and indirectly influenced parents’ mental health [26]. In our replication of this model (A), modified to encompass the two Impact dimensions and with the addition of timing variables, we did not find such an effect of genetic diagnosis. Thus timing differences between genetic diagnosis groups may have confounded the previous observation - SNV diagnoses were on average made at an older age due to more recent availability of technology via large-scale genomics research projects and clinical implementation, thus more recently in time with relation to participation in the study. A second explanation could be that the effect of genetic diagnosis type operates indirectly via the developmental characteristics of the diagnosed child. Hence, results may be influenced by the age-related changes in psychopathology observed in children and young people with ID [45], which may be especially prevalent in children with CNV-associated ID [46]. To test this possibility, we explored model B, finding evidence for serial mediation from genetic diagnosis type to Impact and parents’ mental health, via behavioural and physical characteristics. Pathways within the serial mediation model appear to be relatively specific for each genetic diagnosis type: SNV diagnoses within the sample were more likely to be associated with physical disabilities, mediating effects on Impact and EFQ; CNV diagnoses were more likely to be associated with CD/ODD and other EBD, which mediates the indirect effects for this group. Importantly, severity of ID (DQ) did not contribute to Impact appraisal for any genetic diagnosis group, and presence of ASD predicts Impact for all groups (with the possible exception of sex chromosome aneuploidies). Overall, this model suggests that the intrinsic characteristics of children with ID, influenced by their genetic diagnosis, contributes to parents’ appraisal and well-being, alongside important social factors.

Within model B, we found that the current age of the child served as a direct predictor of Impact: home&distress (older child, higher Impact). In contrast, the time since diagnosis negatively predicted Impact:participation (short duration of diagnosis, higher Impact). A potential explanation could be that Impact:home&distress tends to rise with age in families of children with ID, and that adjustment to a recent diagnosis compounds negative Impact:participation appraisal. A limitation of the current study is that we cannot model the age at time of diagnosis together with time elapsed since diagnosis and current age. To resolve this, the contrasting effects of current age and time since diagnosis on different dimensions of Impact require investigation within a longitudinal study. An additional time-related factor that could not be analysed in this study is the duration of the “diagnostic odyssey” i.e. time between first recognition of a child’s neurodevelopmental differences and receiving a genetic diagnosis. Moreover, a future study should include families who receive a genetic diagnosis before their child is age 3 years, since early diagnosis may have particularly significant impacts for parental adjustment. Comparison to parents of children and young people with neurodevelopmental disorders of non-genetic or unknown cause will also be informative.

To build on the observed associations, we conducted an exploratory analysis to determine whether relationships between genetic diagnosis, child characteristics and parent well-being were moderated by duration of diagnosis. We found initial evidence for ‘fading’ effects of genetic diagnosis type, irrespective of the chronological age of the child at time of participation. This provides some preliminary evidence that, in the short term after diagnosis, provision of diagnosis-specific information and personalised management may have benefits for a family, beyond the hoped-for benefits for the child with ID. However, the observed effects are complex to interpret, especially within cross-sectional data. The greater likelihood of CD/ODD within the CNV group and its relationship with Impact appears to be constant across time. Physical disability differences between groups were large for recently diagnosed children, but minimal for more distantly diagnosed children– this could reflect actual improvements in health for the SNV group and decline in the CNV group or be an artefact of the characteristics of children diagnosed via sequencing historically versus more recently. Whilst the recently diagnosed CNV group had higher EBD difficulties than recently diagnosed SNV group, genetic diagnosis groups did not differ on this variable for historically diagnosed groups. This potentially indicates developmental improvement of the CNV group, but also indicates that emotional and behavioural challenges are important across the whole ID population and require long-term comprehensive support.

## Conclusion

The current analysis found that diverse factors influence variation in parents’ well-being in the context of childhood ID. Whilst large-scale data and SEM analysis methods can identify group-wide predictors and pathways, ultimately each family is unique and complex. Future studies should explore the identified pathways in more detail, via more sophisticated evaluations of genomic variants, child characteristics and parents’ psychology. Crucially, longitudinal studies are essential to move beyond cross-sectional observations and identify interactive, dynamic processes which could be harnessed within routine post-diagnostic care and focused interventions.

### Electronic supplementary material

Below is the link to the electronic supplementary material.

**Supplementary Material 1:** Supplementary tables and figures

## Data Availability

Data sharing for the analyses presented in this paper was approved by the IMAGINE-ID data access committee: https://imagine-id.org/healthcare-professionals/datasharing.
