# Supplementary material for: Rare neurodevelopmental conditions and parents’ mental health – how and when does genetic diagnosis matter?
Source: Orphanet J Rare Dis. 2024 Feb 15;19:70. doi: 10.1186/s13023-024-03076-2 (PMC10870533; doi:10.1186/s13023-024-03076-2)
Supplement: Supplementary file 1 — Supplementary Material 1: Supplementary tables and figures [file 13023_2024_3076_MOESM2_ESM.docx]

Supplementary Material A: Data Cleaning Flow Chart

Original

N=4071

RQ1

N=2655

Combined same child’s genetic variants (duplicate n=661)

Identified and selected siblings (n=81)

filter age ≤ 18 (n=157, missing = 517)

RQ2

N=2423

Filter cases with valid time since diagnosis (Time since Diagnosis > 0)

RQ3

N=1943

Select only CNV and SNV groups

Note:

RQ = Research Question:

RQ1 Factor structure of SDQ Impact scale

RQ2 SEM pathway analyses (models A and B)

RQ2 Moderation of mediating pathways by time since genetic diagnosis

Supplementary Material B. Descriptive Analyses and Group Comparison by Genetic Variant Type

|  | Group | Whole sample | CNV  N = 1525 | SNV  N = 583 | Multiple CNV  N = 271 | Other Chrom  N = 108 | Sex Chrom  Aneuploidy  N = 93 | CNV&SNV  N = 18 | Multiple SNV  N = 57 | Between-Groups Difference | |
| --- | --- | --- | --- | --- | --- | --- | --- | --- | --- | --- | --- |
| Continuous variables |  | Mean (SD) | Mean (SD) | Mean (SD) | Mean (SD) | Mean (SD) | Mean (SD) | Mean (SD) | Mean (SD) | ANOVA | p |
|  | Age | 9.09 (3.82) | 8.63 (3.68) | 10.29 (3.85) | 8.56 (3.67) | 9.21 (4.07) | 9.66 (4.16) | 9.39 (4.43) | 10.53 (3.69) | 16.33 | **<.001** |
|  | DQ | 0.53 (0.26) | 0.56 (0.26) | 0.44 (0.26) | 0.56 (0.24) | 0.45 (0.26) | 0.64 (0.19) | 0.43 (0.27) | 0.39 (0.26) | 19.5 | **<.001** |
|  | Physical | 2.5 (1.92) | 2.23 (1.78) | 3.18 (2.07) | 2.41 (1.74) | 2.79 (2) | 1.66 (1.75) | 3.4 (2.03) | 3.38 (2.34) | 21.8 | **<.001** |
|  | Impact | 5.43 (3.01) | 5.55 (2.95) | 5.28 (3.16) | 5.51 (2.85) | 4.53 (2.86) | 5.24 (3.3) | 6.06 (3.1) | 5.04 (3.46) | 2.63 | 0.02 |
|  | Life events | 0.81 (1.03) | 0.82 (1.05) | 0.78 (0.97) | 0.83 (1.02) | 0.91 (1.01) | 0.69 (0.92) | 1.12 (1.27) | 0.74 (1.18) | 0.85 | 0.53 |
|  | IMD | 5.61 (2.92) | 5.28 (2.9) | 6.26 (2.84) | 5.43 (3.01) | 6.52 (2.68) | 6.27 (2.74) | 5.53 (2.9) | 5.55 (2.74) | 10.35 | **<.001** |
|  | EFQ | 16.79 (7.34) | 16.78 (7.48) | 17.05 (7.29) | 16.37 (6.98) | 15.77 (6.02) | 17.31 (6.81) | 16.63 (7.27) | 17.78 (8.99) | 0.82 | 0.55 |
|  | Time since genetic diagnosis | 2.73 (3.02) | 2.72 (2.95) | 2.2 (2.75) | 2.69 (2.32) | 5.61 (4.37) | 4.34 (4) | 2.34 (2.01) | 1.09 (1.65) | 26.06 | **<.001** |
|  | Age at genetic diagnosis | 6.3 (4.01) | 5.9 (3.75) | 8.05 (4.16) | 5.89 (3.51) | 3.52 (4.07) | 4.74 (4.44) | 6.55 (3.67) | 9.2 (3.7) | 37.87 | **<.001** |
| Categorical variables |  | N (%) | N (%) | N (%) | N (%) | N (%) | N (%) | N (%) | N (%) | Chi^2^ | p |
| Gender | Male | 1456 (0.55) | 881 (0.58) | 289 (0.5) | 147 (0.54) | 51 (0.47) | 41 (0.44) | 9 (0.5) | 38 (0.67) | 22.13 | **0.001** |
|  | Female | 1199 (0.45) | 644 (42.23) | 294 (0.5) | 124 (0.46) | 57 (0.53) | 52 (0.56) | 9 (0.5) | 19 (0.33) |  |  |
| ASD likelihood binary | Yes | 454 (0.17) | 262 (0.17) | 104 (0.18) | 42 (0.16) | 17 (0.16) | 9 (0.1) | 6 (0.33) | 14 (0.25) | 12.11 | 0.06 |
|  | No | 1966 (0.74) | 1141 (0.75) | 406 (0.7) | 210 (0.77) | 83 (0.77) | 80 (0.86) | 12 (0.67) | 34 (0.6) |  |  |
| CD/ODD likelihood binary | Yes | 458 (0.17) | 291 (0.19) | 67 (0.11) | 52 (0.19) | 10 (0.09) | 26 (0.28) | 3 (0.17) | 9 (0.16) | 30.23 | **<.001** |
|  | No | 2197 (0.83) | 1234 (0.81) | 516 (0.89) | 219 (0.81) | 98 (0.91) | 67 (0.72) | 15 (0.83) | 48 (0.84) |  |  |
| Other emotional or behavioural likelihood binary | Yes | 609 (0.23) | 382 (0.25) | 103 (0.18) | 73 (0.27) | 15 (0.14) | 23 (0.25) | 4 (0.22) | 9 (0.16) | 22.29 | **0.001** |
|  | No | 2046 (0.77) | 1143 (0.75) | 480 (0.82) | 198 (0.73) | 93 (0.86) | 70 (0.75) | 14 (0.78) | 48 (0.84) |  |  |
| More than one adult in household | Yes | 1217 (0.88) | 649 (0.86) | 331 (0.91) | 129 (0.92) | 46(0.88) | 21 (0.78) | 10 (0.83) | 31 (0.94) | 13.73 | 0.03 |
|  | No | 165 (0.12) | 107 (0.14) | 31 (0.09) | 11 (0.08) | 6 (0.12) | 6 (0.22) | 2 (0.17) | 2 (0.06) |  |  |
| More than one child in household | Yes | 950 (0.68) | 531 (0.70) | 227 (0.63) | 109 (0.77) | 34 (0.64) | 18 (0.67) | 9 (0.75) | 22 (0.67) | 12.24 | 0.06 |
|  | No | 437 (0.32) | 228 (0.30) | 135 (0.37) | 32 (0.23) | 19 (0.36) | 9 (0.33) | 3 (0.25) | 11 (0.33) |  |  |
| Note: threshold of significance is defined as .003, corrected for multiple comparisons. Significant p values are shown in bold. | | | | | | | | | | | |

Supplementary Material C. SDQ Impact Items and Polychoric Correlation Between Items


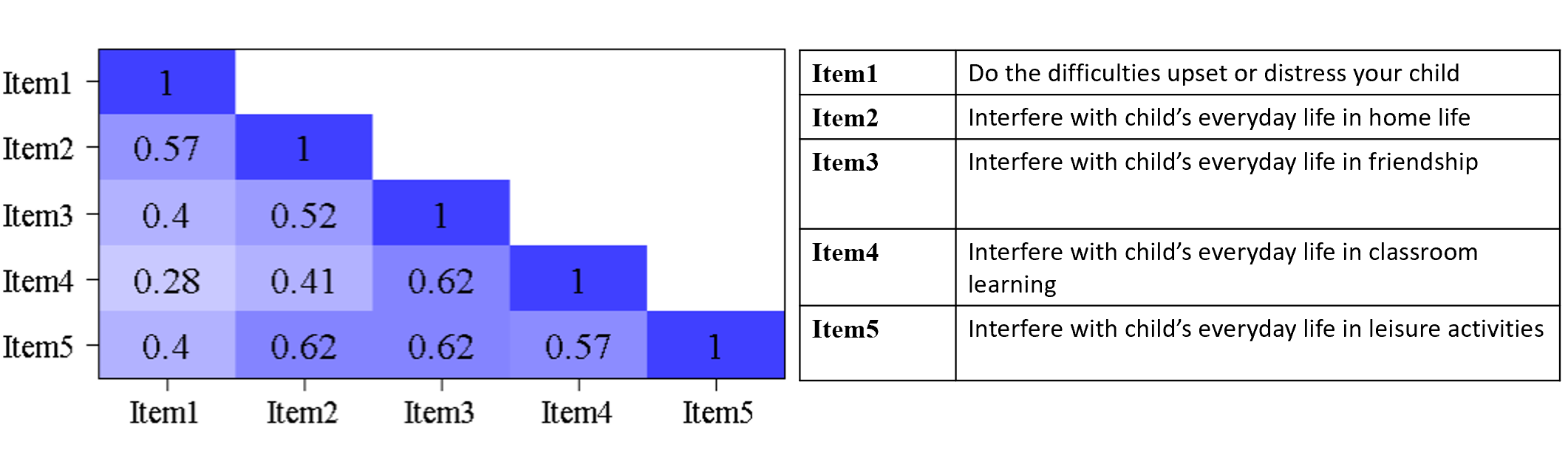


*Note: Polychoric correlations were calculated as all items are ordinal. Colour of the cell indicates significance of correlations (darker = stronger correlation).*

Supplementary Material D: Parallel analysis scree plot

*
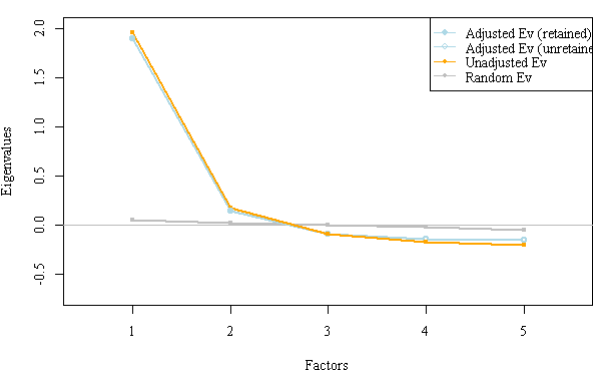
*

Supplementary Material E: Pathway Model A


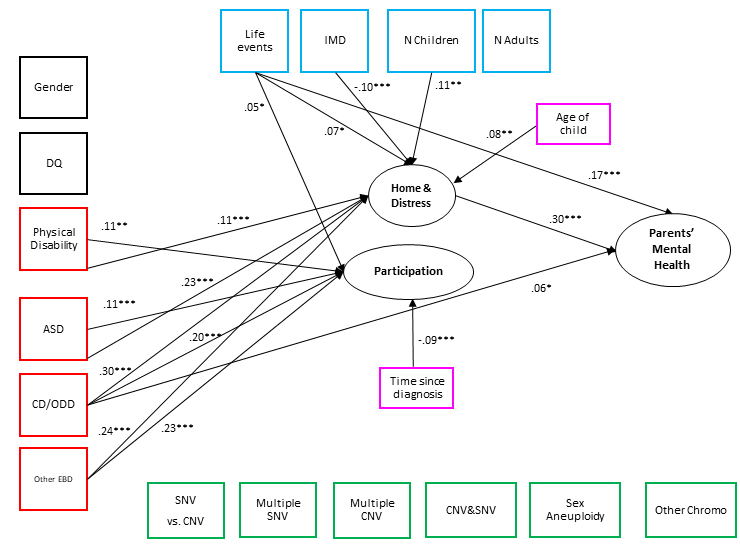


DQ = developmental quotient, ASD = autism spectrum disorder likelihood, CD/ODD = conduct disorder or oppositional defiant disorder likelihood, Other EBD = other emotional or behavioural disorder likelihood, IMD = Index of Multiple Deprivation decile score, N Children = whether more than one child in household, N Adults = whether more than one adult in household. ** p<0.01, *** p<0.001.

Supplementary Material F: Pathway Model B


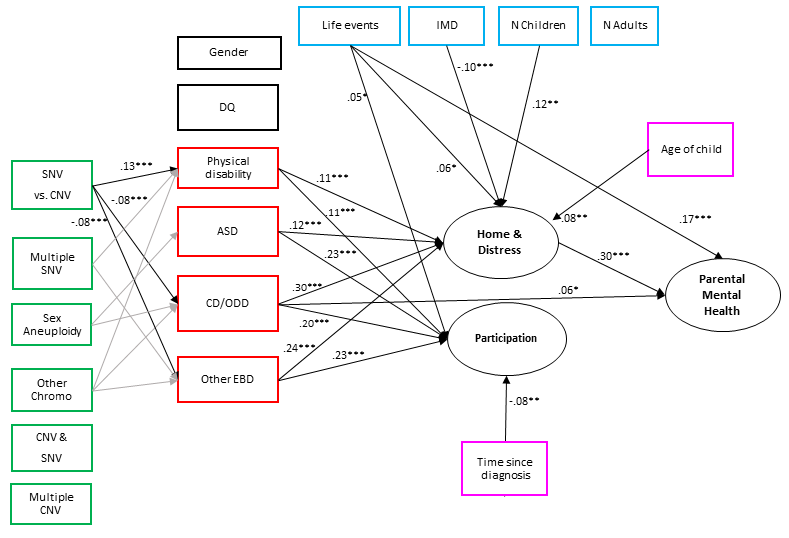


DQ = developmental quotient, ASD = autism spectrum disorder likelihood, CD/ODD = conduct disorder or oppositional defiant disorder likelihood, Other EBD = other emotional and behavioural disorder likelihood, IMD = Index of Multiple Deprivation decile score, N Children = whether more than one child in household, N Adults = whether more than one adult in household. Effect of all genetic diagnosis types were tested (vs. CNV) but due to limitation of sample sizes in each group, only the effect size of SNV vs. CNV is given. Other significant effects are indicated via grey arrows. ** p<0.01, *** p<0.001.

Supplementary Material G: Moderation model


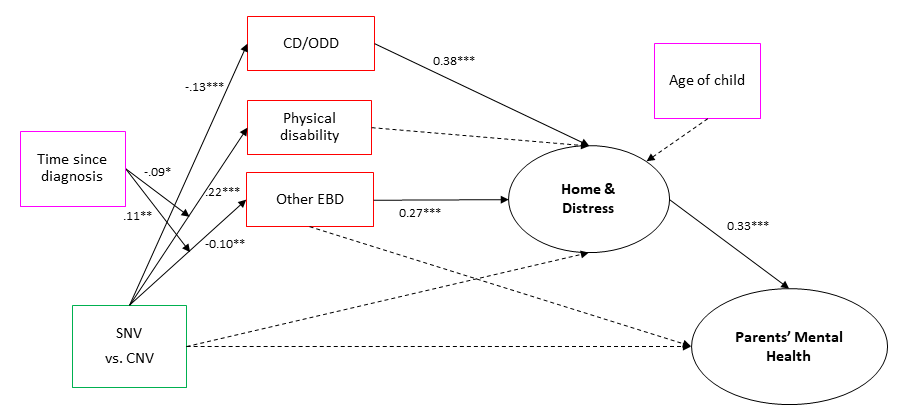


CD/ODD = conduct disorder or oppositional defiant disorder likelihood, Other EBD = other emotional or behavioural disorder likelihood. Pathways that are not significant are shown in dashed lines. ** p<0.01, *** p<0.001.

IMAGINE-ID consortium members

| Surname | Initials | First Name | Title | Institution |
| --- | --- | --- | --- | --- |
| Raymond | F L | F Lucy | Professor | Department of Medical Genetics, University of Cambridge, UK |
| Dewhurst | E | Eleanor | Mrs | Department of Medical Genetics, University of Cambridge, UK |
| Lafont | A | Amy | Ms | Department of Medical Genetics, University of Cambridge, UK |
| Timur | H | Husniye | Ms | Department of Medical Genetics, University of Cambridge, UK |
| Wicks | F | Francesca | Mrs | Department of Medical Genetics, University of Cambridge, UK |
| Ye | Z | Zheng | Dr | Department of Medical Genetics, University of Cambridge, UK |
| Baker | K | Kate | Dr | Department of Medical Genetics, University of Cambridge, UK |
| Walker | N | Neil | Dr | Department of Medical Genetics, University of Cambridge, UK |
| Wallwork | S | Sarah | Ms | Department of Medical Genetics, University of Cambridge, UK |
| Skuse | D | David | Professor | Great Ormond Street Institute of Child Health, University College London, UK |
| Denaxas | S | Spiros | Dr | Institute of Health Informatics, University College London, London, UK |
| Mandy | W | William | Dr | Division of Psychology & Language Sciences, University College London, UK |
| Wolstencroft | J | Jeanne | Dr | Great Ormond Street Institute of Child Health, University College London, UK |
| Davies | S | Sarah | Ms | Great Ormond Street Institute of Child Health, University College London, UK |
| Erwood | M | Marie | Ms | Great Ormond Street Institute of Child Health, University College London, UK |
| Juj | M | Manoj | Mr | Great Ormond Street Institute of Child Health, University College London, UK |
| Kerry | E | Eleanor | Ms | Great Ormond Street Institute of Child Health, University College London, UK |
| Lucock | A | Anna | Ms | Great Ormond Street Institute of Child Health, University College London, UK |
| Printzlau | F | Frida | Ms | Great Ormond Street Institute of Child Health, University College London, UK |
| Srinivasan | R | Ramya | Dr | Great Ormond Street Institute of Child Health, University College London, UK |
| Walker | S | Susan | Dr | Great Ormond Street Institute of Child Health, University College London, UK |
| Watkins | A | Alice | Ms | Great Ormond Street Institute of Child Health, University College London, UK |
| Coscini | N | Nadia | Dr | Great Ormond Street Institute of Child Health, University College London, UK |
| Fatih | N | Nasrtullah | Mr | Great Ormond Street Institute of Child Health, University College London, UK |
| Nayana | L | Lahiri | Ms | Great Ormond Street Institute of Child Health, University College London, UK |
| Denyer | H | Hayley | Ms | Great Ormond Street Institute of Child Health, University College London, UK |
| Andrews | S | Sophie | Ms | MRC Centre for Neuropsychiatric Genetics and Genomics, Division of Psychological Medicine and Clinical Neurosciences, Cardiff University, UK |
| Chawner | SJRA | Samuel | Dr | MRC Centre for Neuropsychiatric Genetics and Genomics, Division of Psychological Medicine and Clinical Neurosciences, Cardiff University, UK |
| Cuthbert | A | Andrew | Dr | MRC Centre for Neuropsychiatric Genetics and Genomics, Division of Psychological Medicine and Clinical Neurosciences, Cardiff University, UK |
| Challenger | A | Aimee | Ms | MRC Centre for Neuropsychiatric Genetics and Genomics, Division of Psychological Medicine and Clinical Neurosciences, Cardiff University, UK |
| Hall | J | Jeremy | Professor | MRC Centre for Neuropsychiatric Genetics and Genomics, Division of Psychological Medicine and Clinical Neurosciences, Cardiff University, UK |
| Lewis | N | Nicola | Ms | MRC Centre for Neuropsychiatric Genetics and Genomics, Division of Psychological Medicine and Clinical Neurosciences, Cardiff University, UK |
| Owen | MJ | Michael | Professor Sir | MRC Centre for Neuropsychiatric Genetics and Genomics, Division of Psychological Medicine and Clinical Neurosciences, Cardiff University, UK |
| Ray | S | Sinead | Ms | MRC Centre for Neuropsychiatric Genetics and Genomics, Division of Psychological Medicine and Clinical Neurosciences, Cardiff University, UK |
| Sopp | M | Matthew | Mr | MRC Centre for Neuropsychiatric Genetics and Genomics, Division of Psychological Medicine and Clinical Neurosciences, Cardiff University, UK |
| Moss | H | Hayley | Ms | MRC Centre for Neuropsychiatric Genetics and Genomics, Division of Psychological Medicine and Clinical Neurosciences, Cardiff University, UK |
| van den Bree | MBM | Marianne | Professor | MRC Centre for Neuropsychiatric Genetics and Genomics, Division of Psychological Medicine and Clinical Neurosciences, Cardiff University, UK |
| Holmans | P | Peter | Professor | MRC Centre for Neuropsychiatric Genetics and Genomics, Division of Psychological Medicine and Clinical Neurosciences, Cardiff University, UK |
| Bowen | S | Samantha | Ms | MRC Centre for Neuropsychiatric Genetics and Genomics, Division of Psychological Medicine and Clinical Neurosciences, Cardiff University, UK |
| Bradley | K | Karen | Mrs | MRC Centre for Neuropsychiatric Genetics and Genomics, Division of Psychological Medicine and Clinical Neurosciences, Cardiff University, UK |
| Birch | B | Philippa | Ms | MRC Centre for Neuropsychiatric Genetics and Genomics, Division of Psychological Medicine and Clinical Neurosciences, Cardiff University, UK |
| Tong | M | Molly | Ms | MRC Centre for Neuropsychiatric Genetics and Genomics, Division of Psychological Medicine and Clinical Neurosciences, Cardiff University, UK |
| Ford | T | Tasmin | Professor | Department of Psychiatry, University of Cambridge |
| Searle | B | Beverly | Dr | Unique Charity, UK |
| Wynn | S | Sarah | Dr | Unique Charity, UK |
| Robertson | L | Lisa | Dr | Aberdeen Royal Infirmary Genetics Service |
| Berg | J | Jonathan | Dr | Ninewells Hospital Dundee Genetics Service |
| Lampe | A | Anne | Professor | Western General Hospital Edinburgh Genetics Service |
| Joss | S | Shelagh | Dr | Glasgow Genetics Centre, Glasgow |
| Brennan | P | Paul | Dr | Northern Genetics Service, Newcastle |
| Kraus | A | Alison | Dr | Yorkshire Regional Genetics Service - Clinical Genetics |
| Weber | A | Astrid | Dr | Cheshire & Merseyside Regional Genetic Service |
| Rawson | M | Myfanwy | Ms | Manchester Centre for Genomic Medicine |
| Quarrell | O | Oliver | Dr | Sheffield Genetic Services |
| Vasudevan | P | Pradeep | Dr | Leicestershire Genetics Centre, Leicester |
| Harrison | R | Rachel | Dr | Nottingham Regional Genetics Service |
| Williams | D | Denise | Dr | West Midlands Regional Genetics Service, Birmingham |
| Maher | E | Eamonn | Professor | East Anglian Medical Genetics Service, Cambridge |
| Kini | U | Usha | Dr | Oxford Genetics Service |
| Clowes | V | Virginia | Dr | London North West Thames Regional Genetics Service |
| Van Dijk | F | Fleur | Dr | London North West Thames Regional Genetics Service |
| Gurasashvilli | J | Jana | Dr | London North East Thames Regional Genetics Service - Great Ormond Street Hospital, London |
| Mansour | S | Sahar | Dr | London South West Thames Regional Genetics Service, St Georges Hospital, Tooting, London |
| Holder-Espinasse | M | Muriel | Dr | London South East Thames Regional Genetics Service Guy's Hospital, London |
| Watford | A | Amy | Dr | Bristol Clinical Genetics Service, Bristol |
| Rankin | J | Julia | Dr | Peninsula Genetics Service, Exeter |
| Baralle | D | Diana | Dr | Wessex Clinical Genetics Service |
| Procter | A | Annie | Dr | All Wales Regional Genetics Service |
